# Early-Onset Parkinson’s Disease With Multiple Positive Intraoperative Spinal Tissue Cultures for Cutibacterium acnes

**DOI:** 10.7759/cureus.17607

**Published:** 2021-08-31

**Authors:** Mervat Mourad, Tija M Passley, John M Purcell, Joerg R Leheste

**Affiliations:** 1 Clinical Specialties, New York Institute of Technology College of Osteopathic Medicine, Old Westbury, USA; 2 Basic Sciences, New York Institute of Technology College of Osteopathic Medicine, Jonesboro, USA; 3 Biomedical Sciences, New York Institute of Technology College of Osteopathic Medicine, Old Westbury, USA

**Keywords:** parkinson’s disease, cutibacterium acnes, lymphatic pump, neuroinflammation, braak’s hypothesis

## Abstract

An estimated 95-97% of Parkinson’s disease (PD) cases are idiopathic, emphasizing the absence of a clear etiologic linkage for this debilitating, neurodegenerative, and progressive motor disease. Increasing evidence suggests a peripheral disease origin and the gradual transition of a pathological process along the gut-brain axis and olfactory routes into the brain. This disease pattern is reminiscent of an infectious process and suggests the presence of one or multiple infectious agents, such as bacteria, viruses, fungi, or prion-like proteins. This unusual paradigm, known as Braak’s hypothesis, was first described by the scientist who developed the staging standard for cellular PD pathology and. Here, we describe a case where the small, anaerobic, Gram-positive *Cutibacterium acnes* was recurrently isolated from intraoperative spinal tissues in a patient with early-onset PD. *C. acnes* is also the bacterium that we previously isolated from cadaveric PD brain tissue. Both observations are consistent with Braak’s hypothesis underscoring the importance of homeostasis and maintained immune-competence for healthy aging of the body and mind.

## Introduction

Parkinson’s disease (PD) is the most common progressive neurodegenerative movement disorder and affects millions of people worldwide. Its incidence and prevalence vary with age, gender, and ethnicity. The collective prevalence is the highest in North America, Europe, and Australia and the lowest in Asia [1-3]. Aside from a relatively small percentage of familial cases with monogenetic or toxicant etiologies, circumstances of disease onset and progression are poorly understood in the vast majority of cases (95-97%).

The defining cellular neuropathological change in PD is the accumulation of insoluble intraneuronal and intraneuritic fibrillary protein (alpha-synuclein) aggregates, respectively, known as Lewy bodies and Lewy neurites. Their formation has been linked to neuroinflammation and cell death [4]. PD appears to spread through the central nervous system (CNS) along defined routes that correlate with the nature and severity of symptoms [5]. This notion triggered the development of the six-level Braak staging system based on progressive cellular pathology and matching symptoms.

Early on, nonmotor symptoms such as reduced smell sensation, constipation, trouble sleeping, and other neuropsychological deficits dominate. The clinical diagnosis of PD is based on the cardinal motor symptoms seen during the middle stages which include resting tremor, muscle rigidity, and bradykinesia. At this stage, 60-80% of the dopaminergic (DA) nerve terminals in the substantia nigra pars compacta (SNpc) are gone, implying that significant damage has occurred before a conclusive diagnosis can be made. Postural instability and the beginning of dementia are linked to the later stages of PD [6]. A definite diagnosis of PD is often made only after a trial of dopaminergic medications produces symptomatic relief.

A better understanding of the human microbiome as it relates to immune function, well-being, and disease has raised the suspicion of an infectious process underlying PD. Braak and colleagues [5] noticed that the first alpha-synuclein deposits and neuronal lesions associated with PD always start in the same two places: The anterior olfactory nucleus (AON) of the olfactory bulb and the dorsal motor nucleus of the vagus nerve which serves the heart, lungs, and gut. They postulated the transition of one or multiple infectious agents along specific nervous pathways (i.e., olfactory and vagus nerves) from the periphery into the CNS [7]. This concept was further developed into Braak’s dual-pathway hypothesis describing parallel CNS invasion via the enteric and olfactory routes [8-10]. This hypothesis also matches with some of the early-stage PD symptoms, such as reduced smell sensation and constipation [11]. The following report features a case of early-onset PD in which recurring *Cutibacterium acnes* infection of spinal tissue was noted. Whether both could be related is an interesting possibility and consistent with Braak’s hypothesis.

## Case presentation

**Figure 1:**
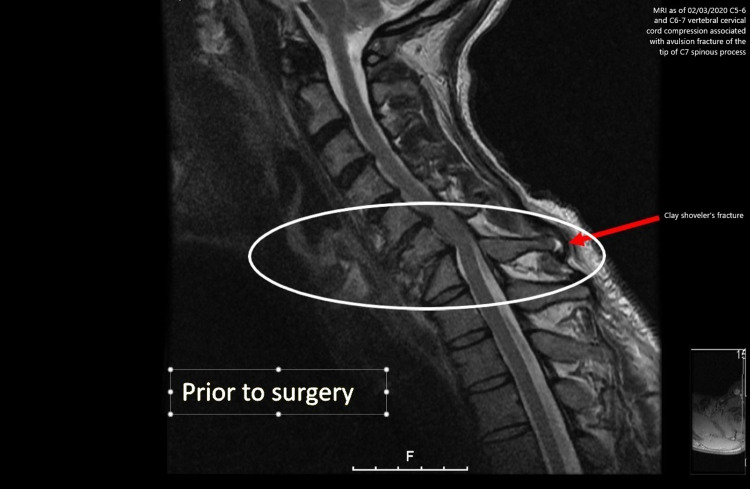
MRI scan indicates C5-6 and C6-7 vertebral cervical cord compression associated with an avulsion fracture of the tip of the spinous process. MRI: magnetic resonance imaging

A 51-year-old male with a medical history of PD diagnosed approximately 15 years ago and degenerative joint disease of the cervical spine (DJD) presented to the local hospital with severe tingling and shooting pain in his neck. Further evaluation with magnetic resonance imaging (MRI) of the cervical spine (with and without contrast) revealed that the DJD was caused by progressive C5-6 and C6-7 endplate irregularities with associated C5-6 and C6-7 vertebral cervical cord compression and an avulsion fracture of the tip of the C7 spinous process known as Clay Shoveler’s fracture (Figure 1). He was then transferred to the hospital for further management.

Three days following the diagnosis, the patient underwent a C5-7 anterior cervical discectomy with anterior interbody arthrodesis and segmental instrumentation with an anterior cervical plate. He underwent harvesting of the iliac crest graft. The surgical drain was removed on postoperative day three. His intraoperative cultures from the spinal tissue grew* C. acnes*, and the University of California San Francisco infectious disease (ID) initially recommended six weeks of intravenous (IV) penicillin G (continuous infusion of 24 million units daily) followed by two weeks of oral amoxicillin (1 g every eight hours) postoperatively. The patient was discharged to acute rehabilitation on postoperative day six with ongoing penicillin IV (same as above) and was instructed to wear a neck brace. He was then transferred to a skilled nursing facility (SNF) on postoperative day 20 on IV ceftriaxone (2 g every 24 hours ) to complete his treatment. A follow-up MRI was done three weeks later which showed interval postoperative changes of the anterior discectomy and fusion at C5 through C7 since the prior MRI study. Mild epidural enhancement was present at these levels, likely postsurgical. No evidence of epidural or paraspinous soft tissue masses or fluid collection was noted.

Against medical advice, he left the SNF two days early and finished his IV treatment successfully and without complications using a home infusion service. The peripherally inserted central catheter was removed by home infusion services. The ID team decided to extend his oral amoxicillin to six months given the indwelling hardware and indolent presentation of his infection. Five months after the initial surgery, he was seen in the ID office complaining of more pain at and below the surgical site and of worsening tremors and/or dyskinesia. At the time, the patient began using a neck brace again after having been off it for a month. He also began having symptoms similar to those prior to the infection such as some discomfort in the anterior clavicular regions. He was taking amoxicillin at the time as recommended by the ID consultant.

**Figure 2:**
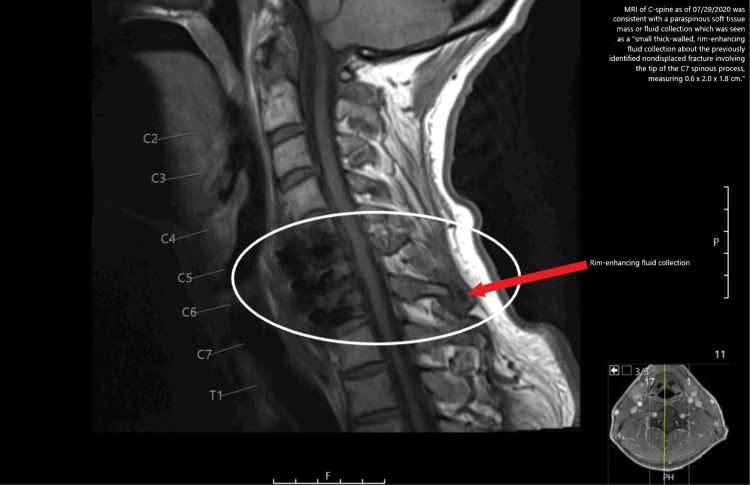
MRI scan of C-spine as of September 29, 2020 indicates a small thick-walled, rim-enhancing fluid collection about the previously identified nondisplaced fracture involving the tip of the C7 spinous process and measuring 0.6 × 2.0 × 1.8 cm. MRI: magnetic resonance imaging

Two weeks later, the patient returned to the emergency department (ED) following an evaluation by urgent care for dizziness and near syncope, which were thought to be medication-related. A diagnostic evaluation in the ED revealed normal vital signs and a mildly elevated white blood cell count with an unremarkable differential (11,800 cells/mm^3^). His C-reactive protein test and sedimentation rate were normal. An MRI of the C-spine on the following day was consistent with intercurrent anterior C5-C7 osteo metallic fusion with nonspecific mild marrow edema and enhancement within the C5, C6, and C7 vertebrae (Figure 2).

Although these findings could solely represent complications of postoperative changes, an infectious origin could not be excluded. There was no well-defined epidural fluid collection/abscess. A paraspinous soft tissue mass or fluid collection was seen as a small thick-walled, rim-enhancing fluid collection about the previously identified nondisplaced fracture involving the tip of the C7 spinous process, measuring 0.6 × 2.0 × 1.8 cm. A small abscess could not be excluded. Procalcitonin levels were assessed as part of an infection workup but were found to be unremarkable. The patient was put back on ceftriaxone IV pending blood cultures. Interventional radiology (IR) was contacted to see if the abscess was amenable to aspiration but the patient opted for outpatient IR drainage. The ID team recommended six months of oral amoxicillin (1 g three times a day) following a course of IV antibiotics due to the foreign bodies (instrumentation) within the infected spine. At the time, a CT-guided fluid aspiration at the C7 spinous process was done and the scant clear liquid was aspirated. It was sent for gram stain and culture. The culture from the aspirate was negative. The patient continued to be on long-term prophylactic antibiotics. The patient reported having mental status changes and/or irregular behavioral tendencies with each episode of infection or neck pain, possibly causing some social difficulties.

Two and a half months later, due to ongoing neck pain and heightened concern, the patient returned to the operating room where his cervical fusion was revised. This included the previously uninvolved C4 and replacement of the anterior cervical plate with a human cadaver chip. Once again, intraoperative cultures from bones and marrow were positive for *C. acnes*. A couple of months following revision surgery, the patient experienced more neck pain. A repeat MRI showed a resurgence of the infection. The patient continues to be under surveillance via repeated application of serial cervical spine MRIs and X-rays. He remains on oral amoxicillin (3 g daily) until now, while blood cultures continue to test negative for bacterial infection.

## Discussion

After decades of intense research, we are still no closer to curing PD. This case presentation intends to reopen the discussion about the possibility of an infectious etiology; in this case, centered on the opportunistic anaerobe *C. acnes*. Currently, there is no hard evidence for causality; however, there are many circumstantial findings, including our own, that may point in that direction. Therefore, it appears prudent to not discount and further investigate Braak’s hypothesis, which is our intention.

One of the most convincing arguments in support of Braak comes from a large (60,000+ per cohort) population-based registry-linkage cohort study in Denmark with individuals who either underwent truncal or superselective (partial) vagotomy between the years of 1977-1995 and follow-up duration of >20 years [12]. Whereas superselective vagotomy did not alter the risk for developing PD, truncal vagotomy significantly reduced the risk. Overall, these findings support Braak’s hypothesis which postulates retrograde pathogen transition along the so-called “gut-brain axis.”

While this model delivers an attractive new perspective on the etiology of PD, information on a potential pathogen or confounding factors remains scarce. Much of the recent research on the pathogenesis of PD focuses on the roles of environmental versus genetic contributors. Both appear to converge at the level of general immune function which is emerging as a significant disease determinant. Several, long-established PD hallmark genes (*SNCA*, *LRRK*, *PINK1*, and *PARKIN*) are now associated with important innate and acquired immune functions, such as cytokine communication, phagocytosis, inflammation, antigen presentation, and general immune barrier maintenance [13]. Natural allelic variations of those genes, mutations, gene duplications, as well as epigenetic changes [14] impact an individual’s PD risk. The same seems to be true for environmental factors such as exposure to immune-compromising insecticides (e.g., pyrethroids), certain viral infections (e.g., influenza, herpes simplex virus 1), as well as the presence of homeostasis-disrupting gut bacteria (e.g., *Heliobacter sp.*) [13]. These works highlight the importance of immune fitness and the ability to establish, maintain, and regulate appropriate immune responses toward the prevention of idiopathic PD. Specific osteopathic manipulative techniques, particularly lymphatic pump techniques (LTPs), are known to augment certain immune functions through lymphatic fluid mobilization [15]. In light of the recently discovered brain lymphatic system (glymphatics), the application of LPTs could provide significant benefits for the augmentation of immune function shielding the CNS from infectious agents [16].

Previous findings from our laboratory are the first to identify the presence of *C. acnes *inside the neurons of human cadaveric PD brain specimen, an observation that could be suggestive of a bacterial connection [17,18]. If this can be further confirmed, it would corroborate Braak’s hypothesis and provide a first glimpse at a specific pathogenic agent. Such confirmation would also shift current perspectives on PD treatment and disease prevention toward immune modulation, possibly through osteopathic techniques, or antibacterial drugs. *C. acnes* is a small Gram-positive anaerobe predominantly found in the pilosebaceous units of the human skin. Until recently, it has been viewed as a mostly benign skin commensal with the potential for triggering acne vulgaris, mostly in adolescents. Driven by improved clinical laboratory culturing techniques for anaerobes, *C. acnes* has come into focus as a potent opportunistic pathogen, especially in postoperative situations [19]. Unlike most bacteria, *C. acnes* has the potential to enter and survive inside the cells of epithelial origin, including neurons. Furthermore, it is known to escape lysosomal breakdown and survive and even multiply inside phagocytes such as neutrophils [17] and macrophages [20].

Recently, we went a step further and tested Braak’s hypothesis in an appropriate animal model. We found strong evidence for the involvement of the nasopharyngeal route in the transition of *C. acnes* into the brain (Leheste, unpublished observations). Whether *C. acnes* actually represents one of the infectious agents Braak postulated remains to be fully determined. The reported case, once again, raises that question which deserves our attention. 

## Conclusions

After decades of research, there is still no cure for the most common neurodegenerative movement disorder on the horizon. Braak’s hypothesis presents an intriguing paradigm centered on an infectious disease etiology for PD that has its beginnings outside of the CNS. This report features a case in which *C. acnes* was recurrently isolated from intraoperative spinal tissues in a patient with early-onset PD. It supports an interesting new perspective on Braak’s hypothesis and the identification of a potential bacterial PD pathogen. This case also warrants a closer look at osteopathic manipulative techniques for the maintenance of immune fitness. Lymphatic techniques are particularly attractive because of the recent discovery of the brain lymphatic system. A better understanding of these connections could be essential for the improvement of PD.
